# Adenovirus vectored IFN-α protects mice from lethal challenge of Chikungunya virus infection

**DOI:** 10.1371/journal.pntd.0008910

**Published:** 2020-12-03

**Authors:** Huixin Chen, Nyo Min, Luyao Ma, Chee-Keng Mok, Justin Jang Hann Chu

**Affiliations:** 1 Laboratory of Molecular RNA Virology and Antiviral Strategies, Department of Microbiology and Immunology, Yong Loo Lin School of Medicine, National University of Singapore, Singapore; 2 Infectious Disease Programme, Yong Loo Lin School of Medicine, National University of Singapore, Singapore; 3 Collaborative and Translation Unit for HFMD, Institute of Molecular and Cell Biology, Agency for Science, Technology and Research (A*STAR), Singapore; University of Tennessee Health Science Center College of Medicine Memphis, UNITED STATES

## Abstract

Chikungunya virus (CHIKV) is a mosquito-borne pathogen that is responsible for numerous large and geographical epidemics, causing millions of cases. However, there is no vaccine or therapeutics against CHIKV infection available. Interferon-alpha (IFN-α) has been shown to produce potent antiviral responses during viral infection. Herein we demonstrated the use of an adenovirus-vectored expressed mouse IFN-α (mDEF201) as a prophylactic and therapeutic treatment against CHIKV *in vivo*. 6-day-old BALB/c mice were pre- or post-treated intranasally with single dose of mDEF201 at 5 x 10^6^ PFU per mouse and challenged with lethal dose of CHIKV. Complete survival protection was observed in mice upon a single dose of mDEF201 administration 1 days prior to virus challenge. Viral load in the serum and multiple organs were significantly reduced upon mDEF201 administration in a dose dependent manner as compare with adenovirus 5 vector placebo set. Histological analysis of the mice tissue revealed that mDEF201 could significantly reduce the tissue morphological abnormities, mainly infiltration of immune cells and muscle fibre necrosis caused by CHIKV infection. In addition, administration of mDEF201 at 6 hours post CHIKV challenge also showed promising inhibitory effect against viral replication and dissemination. In conclusion, single-dose of intranasal administration with mDEF201 as a prophylactic or therapeutic agent within 6 hours post CHIKV infection is highly protective against a lethal challenge of CHIKV in the murine model.

## Author summary

Chikungunya has emerged as one of the most important arbovirus diseases of global health significance. Over the last decade, millions of CHIKV infections have been reported in over 80 countries across several continents. However, there is no approved vaccines or specific therapeutics available, development of antiviral strategies against CHIKV infection has become a pressing issue. In this study, the authors examined using 6-day-old BALB/c mice as an animal model to study CHIKV infection. After shown the 6-day-old mice were permissive and susceptible to CHIKV infection, the authors also evaluated the effectiveness of adenovirus-vectored mouse IFN-α (mDEF201) against systemic and lethal challenge of CHIKV infection in 6-day-old BALB/c mice. Single-dose of mDEF201 can protect mice against a lethal challenge of CHIKV. The authors believe that mDEF201 has immense clinical potential as a single dose could protect individuals at high risks during an outbreak. In addition, mDEF201 could also potentially be used to treat CHIKV infections especially during the acute phase.

## Introduction

Chikungunya virus (CHIKV), a member of the *Alphavirus* from the *Togaviridae* family is a virus transmitted primarily by the mosquito vectors *Aedes aegypti* and *Aedes albopictus* [1,2]. The virus is made up of a positive sense, single-stranded genomic RNA of about 11.8Kb encapsulated within a nucleocapsid and further enwrapped by an envelope [3–5]. The disease, first reported in Tanzania in 1952 currently poses a major threat to the public health worldwide due to the expanding geographical distribution of its mosquito vectors and growing global transport network [6]. In recent years, CHIKV outbreaks have spread from its historical regions in Africa, Asia and India to Europe and the Americas because of the widespread distribution of the vectors *A*. *aegypti* and *A*. *albopictus* [7].

A CHIKV infection is characterized by fever, joint pain and swelling, typically within a week of infection [8]. Other common symptoms include headache, muscle pain and rash [8]. Most healthy individuals recover fully with low mortality rates reported to be less than 1 in 1000 [9,10]. However, some individuals suffer from severe and debilitating chronic rheumatic manifestations at their extremities that could persist for months and even years. Studies have shown that arthritis was persistent in more than 50% of the study subjects years after the initial acute infection [11,12]. Given the long-term complications associated with this disease and the lack of approved vaccines or therapeutics, development of antiviral strategies against CHIKV infection has become a pressing issue. Murine models are a powerful resource for studying chikungunya pathogenesis and to evaluating CHIKV vaccines and therapeutics. There are mainly 3 categories of mouse models for acute CHIKV disease: lethal neonatal challenge models, immunocompromised models of lethal disease, and CHIKV arthritis/myositis models [13]. Among the 3 mouse models, neonatal mice models demonstrated good potential to be used as sensitive tools for testing the efficacy of CHIKV-specific polyclonal and monoclonal antibodies (mAbs) or other therapeutics because of their high sensitivity to CHIKV infection [14].

Type 1 interferon (IFN), a broad-spectrum antiviral protein, plays an important role in the early host immune response by activating pathways that lead to the expression of IFN stimulated genes which in turn confer host protection through the inhibition of viral replication. However, intervention with IFN is highly dependent on the timing of administration, as many viruses have developed mechanisms that suppress the host antiviral IFN response following successful viral RNA replication [15]. The 3h short half-life of IFN also hinders its usage as frequent multiple doses are required to maintain its therapeutic levels *in vivo* [16]. To circumvent the rapid decay of IFN, a replication-deficient (deletions of E1 and E3 genes) adenovirus 5 has been developed to deliver and drive the expression of mouse IFN-α gene (subtype 5) to allow the steady production of mouse IFN-α (mDEF201) in mice. This method of maintaining sustained levels of IFN-α has been shown to offer protection to mice when administered up to a week before infection with Western equine encephalitis virus [17] and one day prior to infection with the genetically related Venezuelan equine encephalitis virus [18]. More recent studies highlighted the anti-viral efficacy of mDEF201 across different viral families as it was revealed that mDEF201 also protected mice against SARS virus [19] and enterovirus A71 [20]. Adenovirus-vectored IFN-α together with mAbs also have been shown to be able to rescue Ebola-infected non-human primates when administered after the detection of viremia and symptoms [21]. Another study that investigated the anti-viral activity of mDEF201 against CHIKV demonstrated that localized arthritic symptoms were alleviated when the arthralgia murine model, DBA/1J mice were administered mDEF201 prophylactically [22]. These data imply that mDEF201 is a promising antiviral candidate especially when used as prophylaxis and to some extent when used during early therapy.

In prospect of the immense potential mDEF201 has as an antiviral agent, we evaluated the effectiveness of mDEF201 in terms of its prophylactic and therapeutic capacity against systemic and lethal challenge of CHIKV infection in 6-day-old BALB/c mice. We investigated the survival rates, viremia, viral load and histology of various organs following CHIKV infection in mice pre- and post-treated with mDEF201 intranasally.

## Materials and methods

### Ethics statement

All animal work were approved and conducted according to the guidelines from the Institutional Animal Care and Use Committee of National University of Singapore (Approved IACUC Protocol No. 023/12).

### Cell lines and virus strain

The cell lines used in this study were BHK (ATCC No. CCL-10) and C6/36 (ATCC No. CRL-1660) mosquito cells. BHK cells were grown in RPMI-1640 medium at 37°C in 5% CO_2_ whereas C6/36 cells were maintained in L-15 medium at 28°C. Both cell lines were grown in T75 cm^2^ tissue culture flasks, supplemented with 10% heat-inactivated FCS. CHIKV LK(EH)CH6708 (GenBank; FJ513654) was kindly provided by the Environmental Health Institute, National Environmental Agency, Singapore.

For generating an infectious CHIKV pool, confluent C6/36 monolayer cells were first infected with CHIKV before maintenance in L-15 medium supplemented with 2% FCS for three to four days. Subsequently, supernatants were harvested and used to determine CHIKV titre by standard viral plaque assays in BHK cells.

### mDEF201 and adenovirus 5 vector

To test for its antiviral efficacy against CHIKV, mDEF201 was kindly provided by Jane Ennis and Jeffrey D. Turner (Defyrus Inc. Toronto, Canada) with a stock titer of 1.45 x 10^9^ PFU/mL. For the test control, the adenovirus 5 vector (Ad5) was prepared as described previously [19]. mDEF201 and Ad5 were diluted with PBS and 3.5 μL was inoculated into 6-day-old mice intranasally.

### mIFN-α quantification by ELISA

5 x 10^6^ PFU of mDEF201 was administered intranasally into 5-day-old BALB/c mice. Ad5 vector was administered at the same dose in the control group. The blood was collected at 6 hours, 1 day, 2 days, 3 days and 7 days post treatment. Serum was separated and the concentrations of mIFN-α were quantified using ELISA (Cat. BMS6027, Invitrogen, USA).

### Establishment of a murine model for CHIKV infection

The animals were housed and experiments were performed in a pathogen-free Animal Biosafety Level 2 (ABSL-2) facility in vivarium in National University of Singapore. 6-day-old BALB/c mice were used in this study. In order to determine the permissibility of 6-day-old BALB/c mice to CHIKV infection, the mortality rate of CHIKV-infected mice was analyzed. The animals were first infected by CHIKV via the intraperitoneal route at 5 x 10^5^ PFU per mouse, followed by daily monitoring over a period of 14 days post-infection. The mice were assessed daily by a mouse clinical scoring system to determine the severity of clinical symptoms of the CHIKV infection (S1 Table). Four criteria which include activity, breathing, movement and change in body weight were used to assess the clinical symptoms in individual mouse and scores were given for each of these criteria. The scores of the four criteria were then summed up to determine the total score. A total score of 6 was defined as the humane endpoint. The number of survivors was recorded daily for two weeks post infection and survivors were euthanized humanely at the end of the experiment. Each mouse was infected with 5 x 10^5^ PFU of CHIKV. They were then sacrificed at 6 hours and 1 to 5 days post infection (dpi) on daily basis. Serum, spleen, brain, liver and limbs were then harvested. Clarified supernatant for downstream viral plaque assay was acquired by homogenizing the harvested tissues with PRECELLYS® beads and centrifuging them at 14,000 g for 10 min.

### Evaluation of the antiviral effects of mDEF201

To investigate the dose dependent antiviral effects of mDEF201, mice were administered with mDEF201 intranasally at varying doses of 5 x 10^4^ PFU per mouse, 1 x 10^5^ PFU per mouse, 2 x 10^5^ PFU per mouse, 5 x 10^5^ PFU per mouse, 5 x 10^6^ PFU per mouse. 5 x 10^6^ PFU per mouse of mDEF201 was used for prophylactic and therapeutic effects studies subsequently. For all studies, 5 x 10^6^ PFU per mouse of Ad5 was utilized as vector controls.

For both dose dependent treatment and prophylactic treatment studies, mDEF201 was administered intranasally before CHIKV was injected intraperitoneally 24 h later. In contrast, mice were first infected with CHIKV for the therapeutic study. mDEF201 was then administered at different time points of 6 h, 12 h and 24 h post CHIKV challenge. The mortality of CHIKV infected mice was assessed by recording the number of survivors daily for 14 days post-infection. All surviving mice were euthanized humanely afterwards.

To investigate the viremia and viral load in the CHIKV infected mice tissues, the animals were sacrificed at 48 h for dose dependent and therapeutic studies. In prophylactic study, the mice tissues were harvested from 6 hours to 5 days post infection on daily basis. Serum, spleen, brain, liver and limbs were harvested, and supernatant was prepared as described above. For the histopathology study, mice were sacrificed 7 dpi.

### Histopathological analysis

The spleen, brain, liver and limbs were harvested and fixed with 4% paraformaldehyde for a week at 4°C before decalcification for two hours at room temperature. The tissues were then embedded in paraffin before being sectioned in thickness of 4 μm. Finally, tissue sections were stained with hematoxylin and eosin (H&E) and evaluated by light microscopy to assess the extent of tissue damage. H&E-stained sections were scored for histopathological evidence. The severity of damage in the brain tissues were scored using a 0–4 point scale, in which 0 = no damage, 1 = mild, 2 = moderate, 3 = severe and focal, and 4 = severe and diffuse. The liver and spleen samples were scored base on the extent of necrosis, with 0 = none, 1 = mild, 2 = moderate, and 3 = severe. The limb muscle samples were scored according to the degree of inflammation and infiltration: 0 = no inflammation, 1 = minimal inflammatory infiltration, 2 = mild infiltration, 3 = moderate infiltration with moderate edema, 4 = severe infiltration with edema. Values represent the mean ± SEM. P values were determined using the Student's t-test.

### Viral plaque assay

To determine the viremia in mice serum and viral load in the homogenized tissues, viral plaque assay was carried out. BHK cells seeded at 90% confluency on 24-well plate were infected by the clarified supernatant obtained from the previously prepared homogenized mice tissue. Infected BHK cells were incubated in an overlay media (1% carboxymethyl cellulose and RPMI-1640 media supplemented with 2% FCS) at 37°C in 5% CO_2_. Three days after CHIKV infection, the cells were stained with crystal violet dye and the number of plaques was then counted to quantify the infectious virus titre.

### Recombinant mIFN-α protein treatment in vivo

6-day-old BALB/c mice were infected with 5 x 10^5^ PFU of CHIKV followed by daily treatment with 100 U/g of recombinant mIFN-α proteins (Gibco, USA) intraperitoneally starting from 6 hpi or 12 hpi to 7 dpi (n = 8 mice per group). Mice were monitored daily over a period of 14 days post-infection and were assessed by a mouse clinical scoring system to determine the severity of clinical symptoms of the CHIKV infection (S1 Table). Four criteria which include activity, breathing, movement and change in body weight were used to assess the clinical symptoms in individual mouse and scores were given for each of these criteria. The scores of the four criteria were then summed up to determine the total score. A total score of 6 was defined as the humane endpoint. The number of survivors was recorded daily for two weeks post infection and survivors were euthanized humanely at the end of the experiment.

## Results

### mDEF201 produces abundant mIFN-α in mice

**Fig 1:**
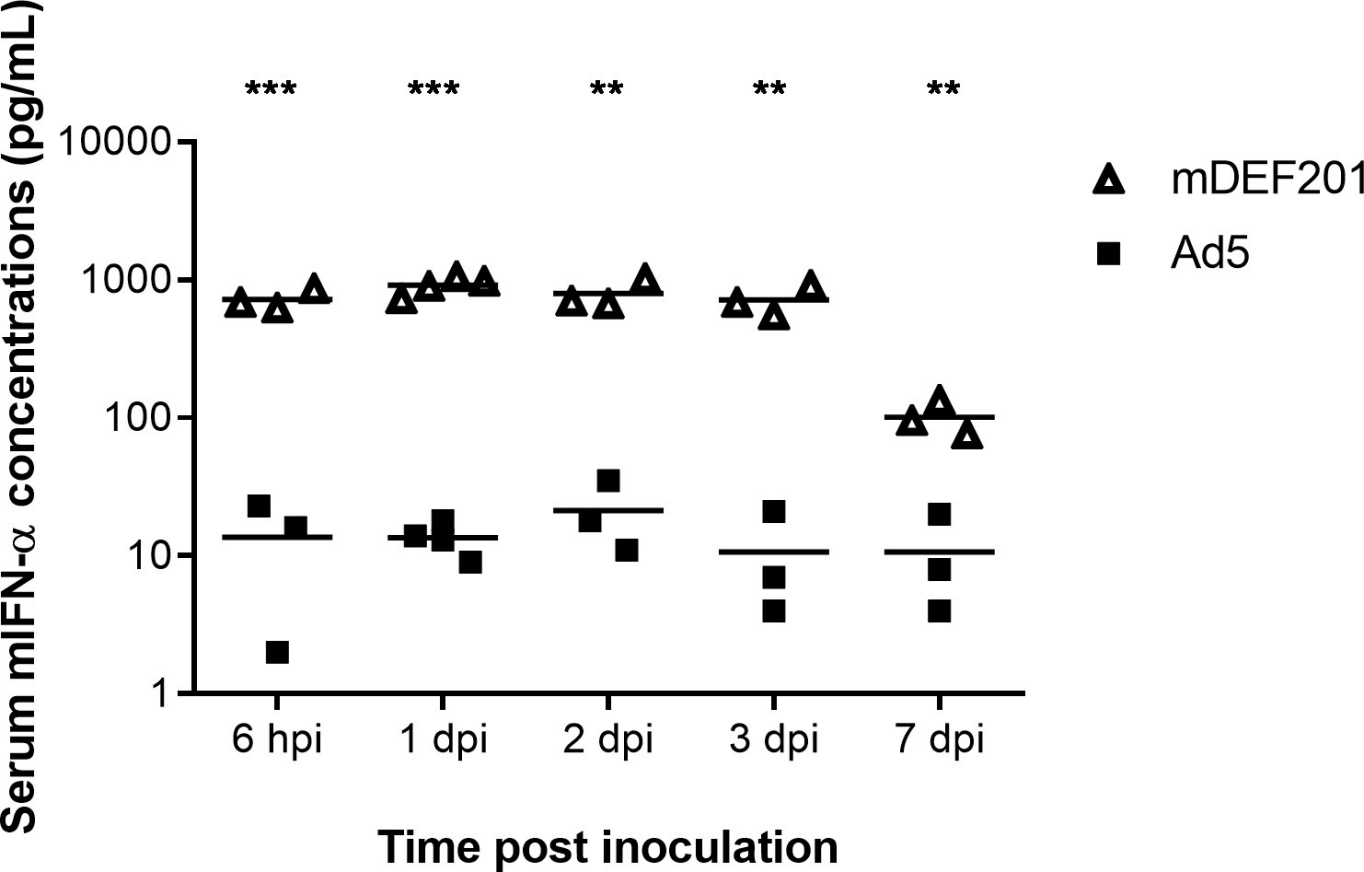
mIFN-α expression driven by mDEF201 *in vivo*. 5-day-old BALB/c mice were administered intranasally with 5 x 10^6^ PFU of mDEF201 or Ad5, and serum mIFN-α concentration was measured at different time points by ELISA. Values presented as mean and individual values from 3 to 4 mice. P values were determined using the Student's t-test. *P < 0.05; **P < 0.01; ***P < 0.001.

The expression of mIFN-α driven by mDEF201 was examined by ELISA. 5-day-old BALB/c mice were inoculated with 5 x 10^6^ PFU of mDEF201 intranasally. Serum was collected at different time points after inoculation and measured for the concentration of mIFN-α. Abundant mIFN-α (722.3 ± 71.2 pg/mL) was detected in mice serum as early as 6 hours post inoculation and remained consistently high at 1, 2, 3 and 7 days post inoculation (Fig 1). No mIFN-α was produced in mice inoculated with the Ad5 vector control. These data demonstrate that a single inoculation of mDEF201 is able to stimulate a rapid and continuous production of mIFN-α.

### Murine model for CHIKV infection

**Fig 2:**
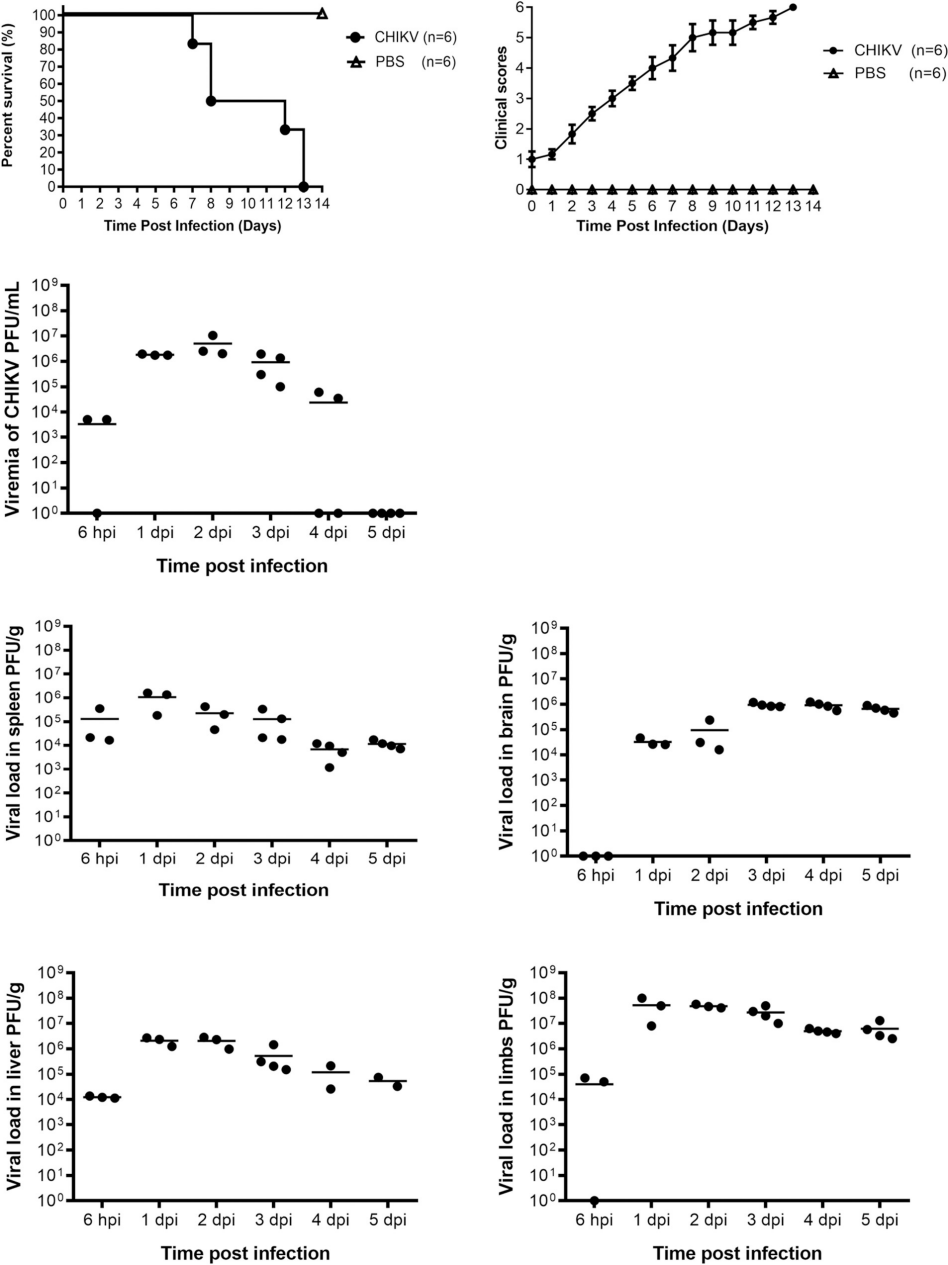
CHIKV infection and replication in mice. (A) 6-day-old BALB/c mice were infected with 5 x 10^5^ PFU CHIKV intraperitoneally. Survival was monitored daily for 14 days and displayed as Kaplan-Meier survival curves (n = 6 per group). (B) Mice were scored based on activity, breathing, movement and body weight change daily (S1 Table). (C to G). Serum, spleen, brain, liver and limbs (n = 3 to 4 mice per group) were harvested at 6 hpi, 1 dpi, 2 dpi, 3 dpi, 4 dpi and 5 dpi and the amount of infectious virus particles were quantified by plaque assay. Values presented as mean and individual values from 3 to 4 mice.

CHIKV was injected at an infective dose of 5 x 10^5^ PFU into 6-day-old BALB/c mice intraperitoneally and the survival rate, clinical scores, viremia and viral load in various tissues were examined to determine whether CHIKV could establish an infection in these mice. The survival curve was generated by close daily monitoring of the mice for two weeks after infection with 5 x 10^5^ PFU of CHIKV. All mice succumbed to the infection by 13 days post infection as revealed in Fig 2A. The disease severity as quantified by clinical scores is depicted in Fig 2B. The susceptibility of the murine model with CHIKV infection was further confirmed by quantifying the infectious viral particles in the various murine tissues upon infection. CHIKV was detected in the serum 6 hours post infection and viral titre peaked at day 2 post infection. By day 5 post infection the virus was eliminated from the blood (Fig 2C). In contrast, viral load was found to persist in the spleen (Fig 2D), brain (Fig 2E), liver (Fig 2F) and limbs (Fig 2G) until 5 dpi. Infectious CHIKV was detected in the brain from 1 day post infection. From these results, 6-day-old BALB/c mice were shown to be highly permissive and susceptible to CHIKV infection.

### Dose dependent antiviral effects of mDEF201

To evaluate the antiviral effect mDEF201 has against CHIKV, mDEF201 was administered at different doses intranasally into 6-day-old BALB/c mice 24 h prior to CHIKV challenge.

**Fig 3:**
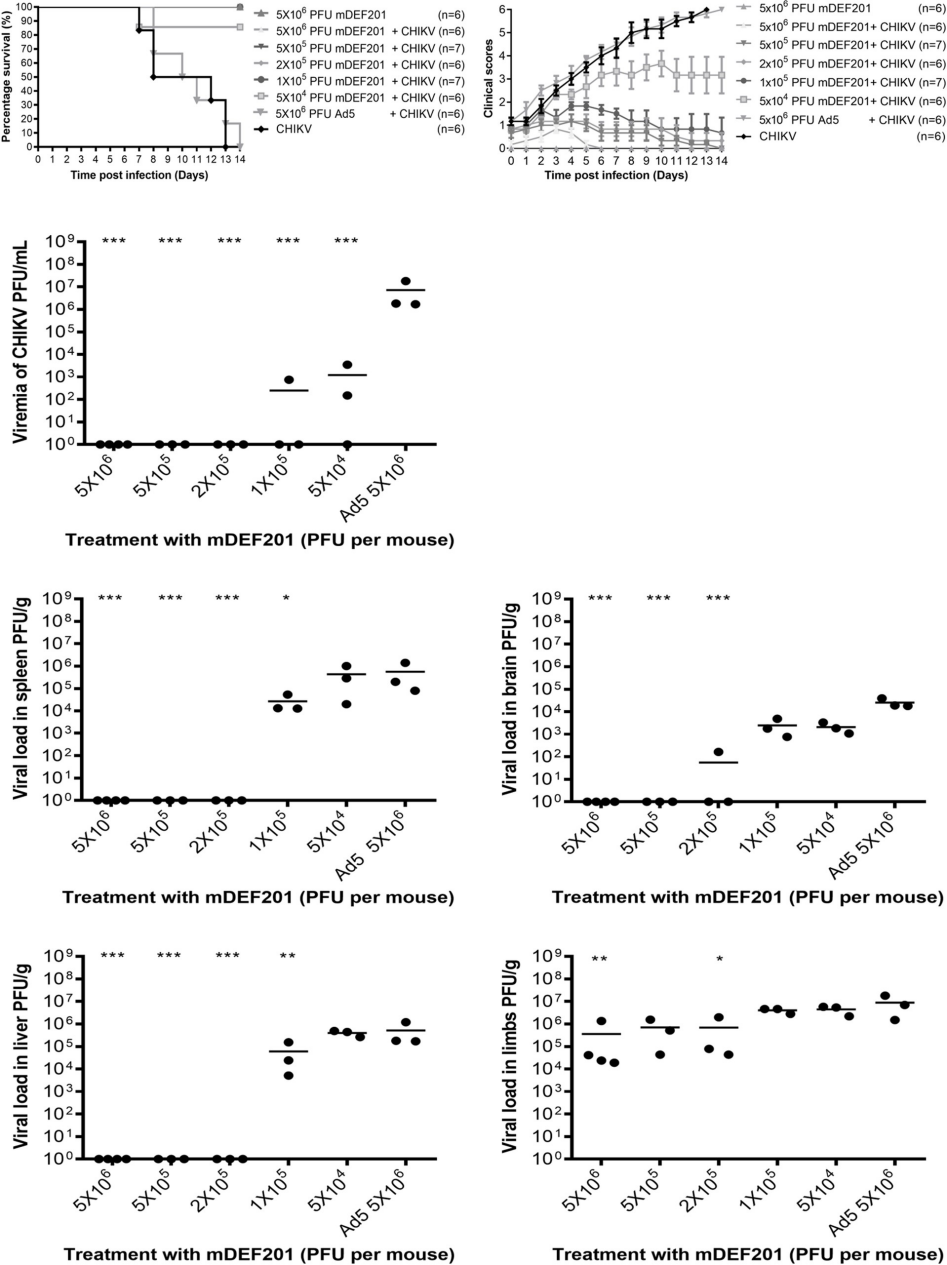
Dose-dependent inhibitory effect of mDEF201 against CHIKV infection. 6-day-old BALB/c mice were administrated intranasally with various doses of mDEF201 and 24 hours later mice were infected with 5 x 10^5^ PFU of CHIKV intraperitoneally. (A) Survival was monitored daily for 14 days and displayed as Kaplan-Meier survival curves. (B) Mice were scored based on activity, breathing, movement and body weight change daily (S1 Table). (C to G) Serum, spleen, brain, liver and limbs (n = 3 to 4 mice per group) were harvested at 2 dpi and the amount of infectious virus particles were quantified by plaque assay. Statistical analysis was carried out using one-way ANOVA test followed by Dunnett’s post-test (compared against Ad5 control). *P < 0.05; **P < 0.01; ***P < 0.001.

The dose dependent relationship between the amount of mDEF201 administered and the mice survival rate was illustrated in Fig 3A. At the treatment dose of 5 x 10^4^ PFU of mDEF201 per mouse, 83% of the mice survived 14 dpi. Higher doses at 1 x 10^5^ PFU of mDEF201 per mouse and higher doses further enhanced the protection against CHIKV infection, with the survival rate similar to the mock-infected mice at 100%. On the other hand, mice pre-treated with the Ad5 vector displayed 100% mortality rate, indicating that the Ad5 vector conferred no protective effect. The disease severity in terms of clinical score is depicted in Fig 3B.

To quantify the infectious CHIKV titre in mice which is pre-treated with mDEF201 followed by infection with CHIKV, serum, spleen, brain, liver and limbs were collected at 2 dpi for plaque assays. As shown in Fig 3C, no virus was detected in the mice serum when they were pre-treated with 2 x 10^5^ PFU of mDEF201 per mouse and higher doses. Pre-treatment with 1 x 10^5^ and 5 x 10^4^ PFU of mDEF201 per mouse significantly reduced virus titre in serum by 4.5 log_10_ and 3.8 log_10_ PFU/g, respectively when compared to mice which were treated with Ad5 vector control. Likewise, no virus was detected in both the spleen and liver when 2 x 10^5^ PFU of mDEF201 per mouse and higher doses were administered before CHIKV challenge (Fig 3D and 3F). A higher dose of mDEF201 (2 x 10^5^ PFU per mouse) was required to completely eradicate the viral load in the brain (Fig 3E). Similar to the spleen and liver, the viral load in the brain is negatively correlated to the dose of mDEF201 administered. However, the virus titre was 5.7 log_10_ PFU/g in the limbs at the highest mDEF201 dose of 5 x 10^6^ PFU of mDEF201 per mouse (Fig 3G). Nevertheless, the viral load was also observed to decrease with increasing mDEF201 doses.

These results showed that mDEF201 indeed offered protection when it is given to mice before CHIKV challenge. The dose dependent prophylactic activity of mDEF201 against CHIKV infection was apparent at lower doses below 1 x 10^5^ PFU of mDEF201 per mouse. 5 x 10^6^ PFU of mDEF201 per mouse was used for all subsequent prophylactic and therapeutic studies to ensure consistency of the data obtained.

### Prophylactic activity of mDEF201

**Fig 4:**
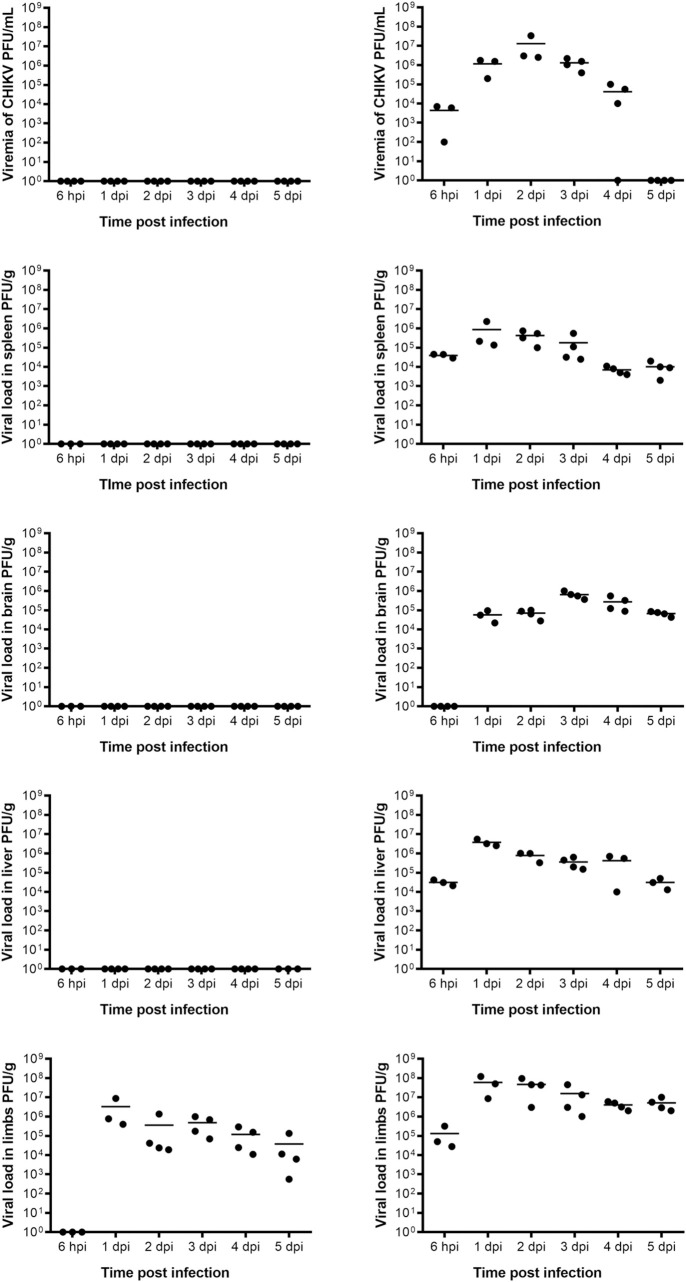
Antiviral effect of mDEF201 against CHIKV. 6-day-old BALB/c mice were pre-treated with 5 x 10^6^ PFU of mDEF201 per mouse 24 h prior to CHIKV infection. Serum, spleen, brain, liver and limbs (n = 3 to 4 mice per group) were harvested at 6 hpi, 1 dpi, 2 dpi, 3 dpi, 4 dpi and 5 dpi, and the amount of infectious virus particles were quantified by plaque assay. Values presented as mean and individual values from 3 to 4 mice.

The prophylactic effect of mDEF201 was investigated by pretreating the mice with 5 x 10^6^ PFU of mDEF201 per mouse 24 h prior to CHIKV infection. The various tissues were harvested on the day of virus inoculation and on a daily basis for 5 consecutive days post infection. Consistent with the dose dependent study, no CHIKV was detected in the mouse serum, spleen, liver and brain of mDEF201 pre-treated, CHIKV infected mice on all days (Fig 4A). In contrast, infectious CHIKV was present at high levels on all days in the serum, spleen, liver and limbs of mice treated with the Ad5 vector (Fig 4B). CHIKV was also noted in the brain at 1 dpi and thereafter in the Ad5-treated mice. In parallel to the dose dependent study, infectious CHIKV was persistently found in the limbs, on all days post infection despite pre-treatment with 5 x 10^6^ PFU of mDEF201 per mouse. However, the viral load in the limbs of these pre-treated mice with mDEF201 was observed to reduce significantly as the infection progresses. Notably, viral load in the limbs was significantly reduced by 1.2 log_10_ PFU/g in the mice pre-treated with mDEF201 as compared to those treated with Ad5 vector on all days post infection.

### Histopathology analysis of the murine tissues

**Fig 5:**
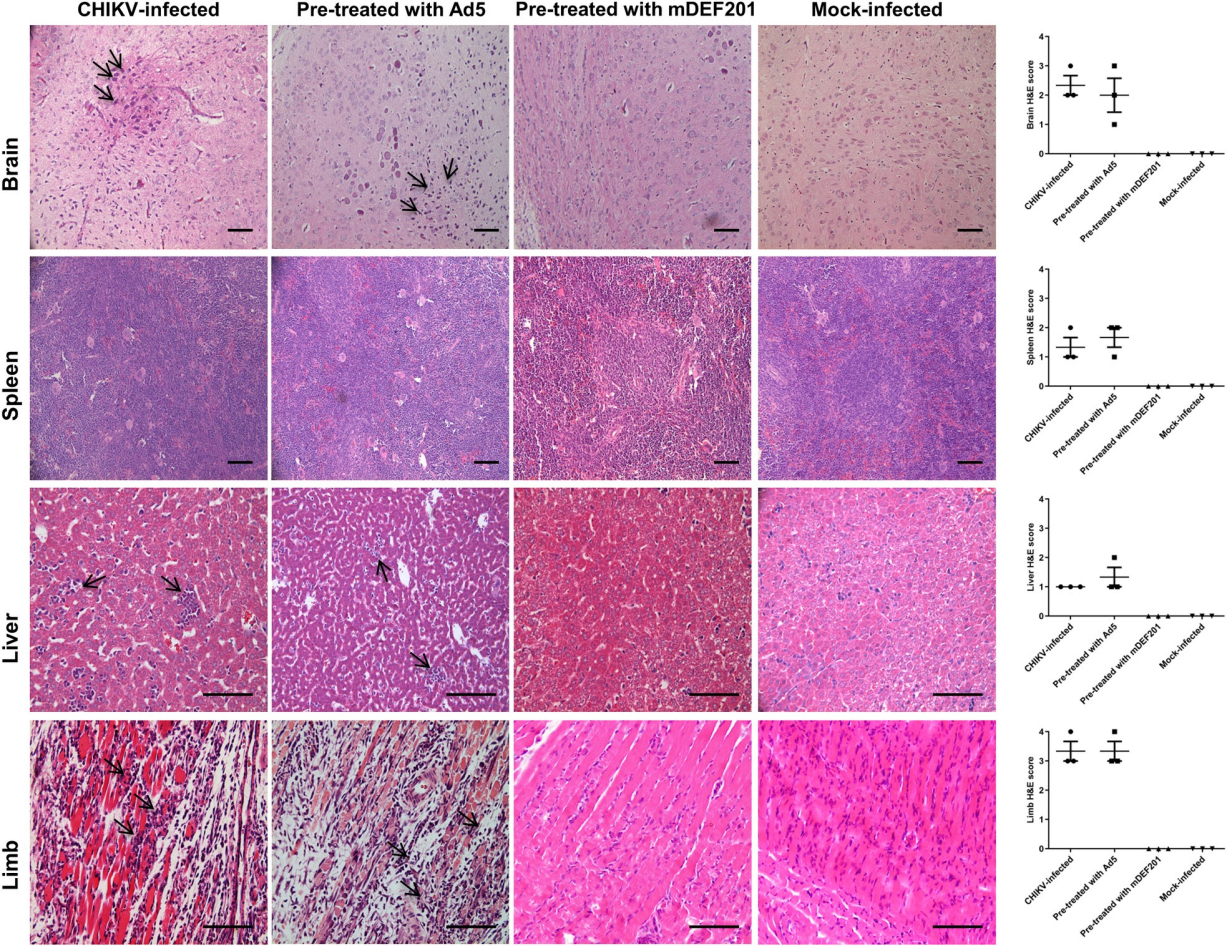
Histopathology analysis of CHIKV challenged mice. 6-day-old BALB/c mice were pre-treated with 5 x 10^6^ PFU of mDEF201 per mouse 24 h prior to CHIKV infection. Brain, spleen, liver and limb muscle were harvested at 7 dpi and stained with hematoxylin and eosin (H&E). Arrows indicate areas of inflammation. Scale bar is 40 μm. H&E-stained sections were scored for histopathological evidence and results are expressed as disease score of tissues. The brain tissues were scored according to the degree of inflammatory infiltrates using a 0–4 point scale, in which 0 = no damage, 1 = mild, 2 = moderate, 3 = severe and focal, and 4 = severe and diffuse. The spleen and liver samples were scored base on the necrosis, with 0 = none, 1 = mild, 2 = moderate, 3 = severe and 4 = very severe. The limb muscle samples were scored according to degree of inflammation and infiltration: 0 = no inflammation, 1 = minimal inflammatory infiltration, 2 = mild infiltration, 3 = moderate infiltration with moderate edema, 4 = severe infiltration with edema. Values present the mean and individual value.

The histopathology analysis of the murine tissue was shown in Fig 5. Mice infected with CHIKV without treatment and mice pre-treated with Ad5 vector followed by infection with CHIKV showed inflammation and infiltration of immune cells in the brain tissues. Mice pre-treated with mDEF201 and infected with CHIKV retained healthy brain tissues similar to the mock-infected mice. Intact white pulp structures were observed in the spleen of mice which is pre-treated with mDEF201 and infected by CHIKV. This is similarly observed in the mock-infected mice. In contrast, the while pulp structures were degenerated when the mice were pre-treated with Ad5 vector followed by infection with CHIKV, and untreated mice. Immune cells infiltration and necrosis were also observed in the liver of mice which were pre-treated with Ad5 vector followed by CHIKV infection, and untreated mice. In contrast, healthy liver morphology was maintained in the mice pre-treated with mDEF201 followed by CHIKV infection, and mock-infected mice. The protective effect of mDEF201 was the most apparent in the muscle tissues of the limbs. Severe tissue damage with loss of muscle fiber, coupled with infiltration of neutrophils and monocytes were evident in the limbs of CHIKV-infected mice which is treated with Ad5 vector or untreated. However, the muscle tissues in mice pre-treated with mDEF201 and mock-infected mice maintained tissue structure integrity with minimal damages. Histopathology scores for tissue necrosis, inflammation and filtration appeared more severe for Ad5 pre-treated and untreated mice than mDEF201 pre-treated mice. Taken together, the above results demonstrated the prophylactic efficacy of mDEF201 against CHIKV infection.

### Therapeutic activity of mDEF201

**Fig 6:**
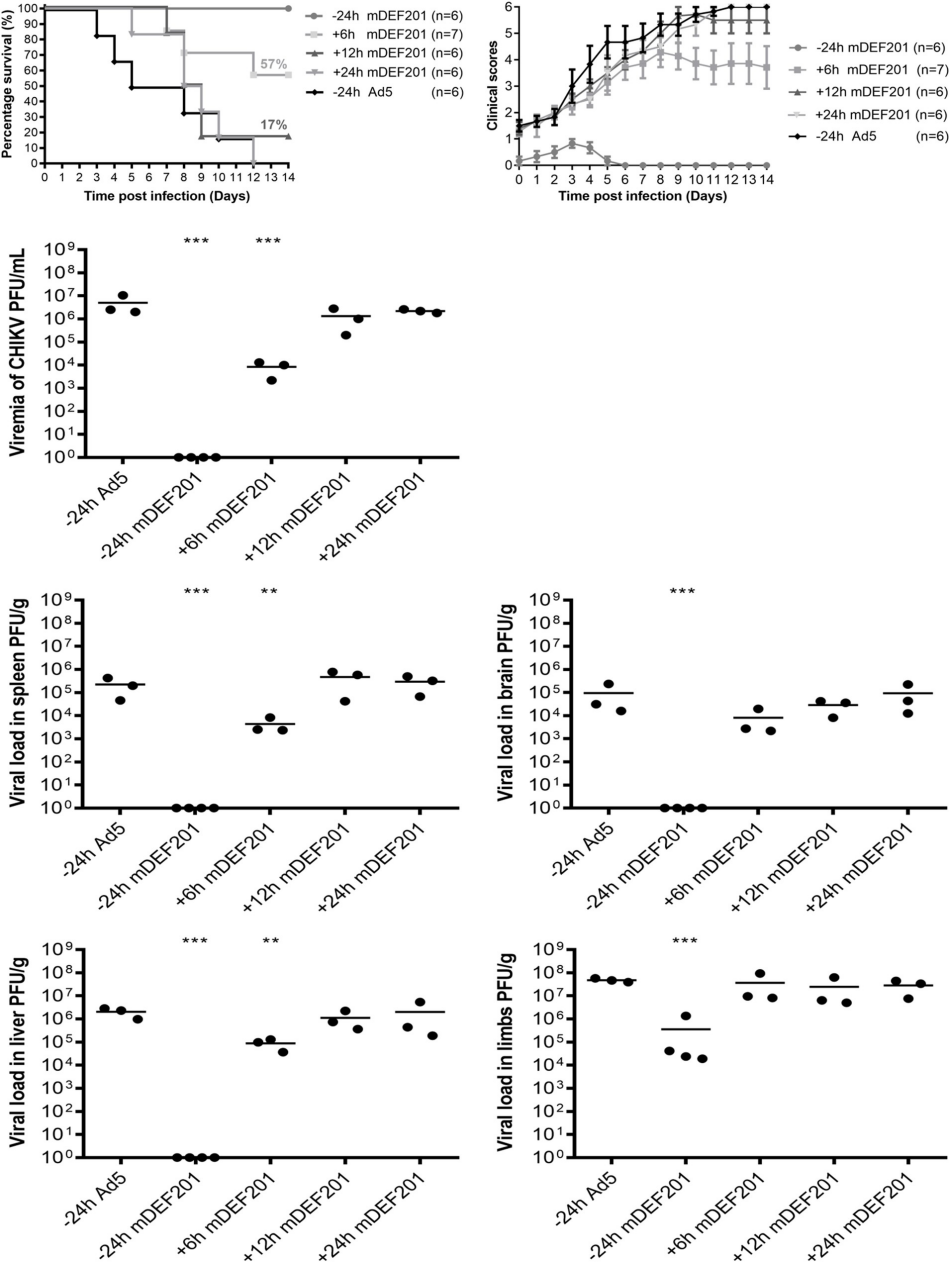
Antiviral efficacy of mDEF201 against CHIKV infection at various times. 6–day-old BALB/c mice were infected with CHIKV at 5 x 10^5^ PFU per mouse and treated with 5 x 10^6^ PFU of mDEF201 per mouse at 6h, 12h or 24h post-infection. The same dose of mDEF201 or Ad5 was administrated 24h before CHIKV infection as treatment controls. (A) Survival of the mice were monitored daily for 14 days. (B) Mice were scored based on activity, breathing, movement and body weight change daily (S1 Table). (C to G) Serum, spleen, brain, liver and limbs (n = 3 to 4 mice per group) were harvested at 2 dpi and the amount of infectious virus particles were quantified by plaque assay. Statistical analysis was carried out using one-way ANOVA test followed by Dunnett’s post-test (compared against Ad5 control). *P < 0.05; **P < 0.01; ***P < 0.001.

In the interest of whether the antiviral activity of mDEF201 extends beyond prophylaxis, post-treatment of mDEF201 following CHIKV infection was performed to examine its therapeutic effect. mDEF201 was administered at 5 x 10^6^ PFU per mouse at different time points after CHIKV infection. The mice pre-treated with mDEF201 24 hours prior to CHIKV challenge served as control. As shown in Fig 6A, the survival rates were 57% and 17% when mDEF201 treatment was administered at 6 hpi and 12 hpi, respectively. The mortality rate was 100% in mice post-treated with mDEF201 24 hpi, or Ad5 vector. Only the mice pre-treated with mDEF201 survived the infection with CHIKV. The disease severity in terms of clinical score is depicted in Fig 6B.

Mice serum, spleen, brain, liver and limbs were harvested 748 hpi and were processed for downstream plaque assays as described previously. From the viremia study (Fig 6C), post treatment with mDEF201 did not completely suppress virus production in the serum unlike those who were pre-treated from which there was no detectable virus. However, the viremia was significantly lower in mice treated at 6 hpi compared to the mock-treated mice. In addition, no significant viremia reduction was observed when mDEF201 was administered at 12 hpi or 24 hpi, demonstrating that the antiviral effects of mDEF201 was lost with time. This time dependent antiviral effect of mDEF201 against CHIKV was found to be similar in the spleen, brain and liver (Fig 6D–6F). Nevertheless, post-treatment with mDEF201 did not appear to reduce viral load in the limbs regardless of the time of administration (Fig 6G).

**Fig 7:**
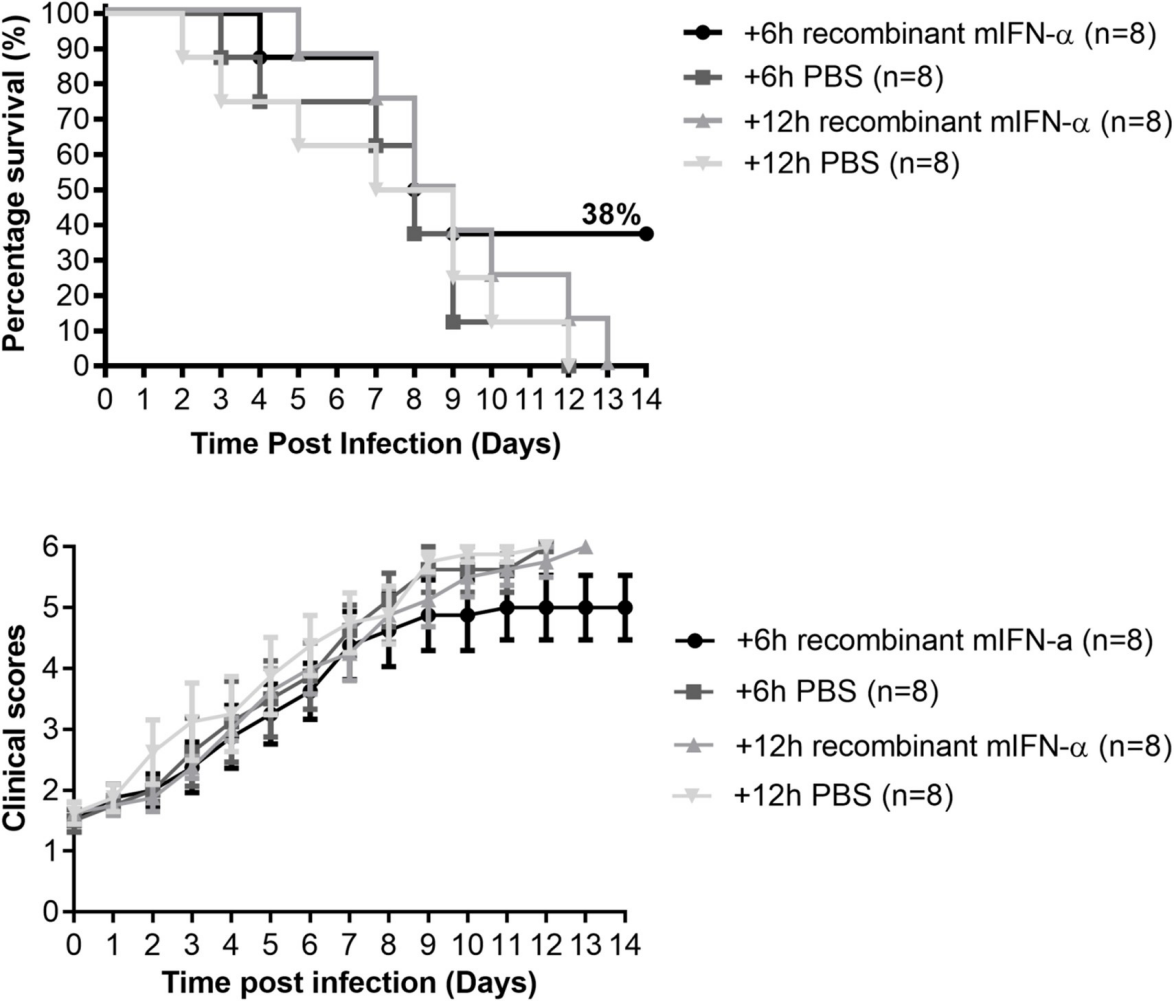
6–day-old BALB/c mice were infected with CHIKV at 5 x 10^5^ PFU per mouse and treated with 100 U/g of recombinant mIFN-α or equal volume of PBS at 6 hpi or 12 hpi to 7 dpi on daily basis (n = 8 mice per group). (A) Survival was monitored daily for 14 days and displayed as Kaplan-Meier survival curves. (B) Mice were scored based on activity, breathing, movement and body weight change daily (S1 Table).

To exclude the possibility that the 57% and 17% survival rates were due to insufficient production of mIFN-α by mDEF201, we administered recombinant mIFN-α protein intraperitoneally starting from 6 hpi or 12 hpi to 7 dpi on a daily basis and monitored the mice survival rate. As shown in Fig 7A, only 38% of the mice survived when recombinant mIFN-α treatment started at 6 hpi and all mice succumbed to CHIKV infection when first treatment was given at 12 hpi. There was no significant improvement in the clinical score of mice which received recombinant mIFN-α treatment 12 hpi compared to the PBS treated group (Fig 7B). Apart from the prophylactic effect mDEF201 has, these data also showed that post treatment with mDEF201 could offer some protection to CHIKV infected mice, especially if administered within 6 hour post CHIKV infection.

## Discussion

A previous study utilized the DBA/1J mouse strain as a non-lethal murine model to specifically replicate CHIKV associated arthralgia that is localized at the limbs [22]. In the study by Dagley and colleague, the amount of footpad swelling, virus titre in the limbs and spleens, and the cytokine profiles of the limbs were studied and the antiviral effects of mDEF201 were characterized [22]. However, the protective effect of mDEF201 on mice challenged with lethal dose of CHIKV infection was not being investigated. Hence, the protective role of mDEF201 during a systemic CHIKV infection is lacking. In this study, BALB/c mice were used to model the systemic infection caused by CHIKV. Apart from the commonly observed arthritic manifestations at the limbs, CHIKV have occasionally been found to disseminate into the central nervous system, causing neurologic complications such as meningitis and encephalitis [23–25]. Therefore, different tissues including the serum, spleen, liver and brain were investigated in this study to thoroughly examine the pathology in a systemic infection.

This study demonstrated that a single prophylactic dose of 1 x 10^5^ PFU of mDEF201 per mouse was able to protect 6-day-old BALB/c mice from a lethal dose of CHIKV infection. This protection against CHIKV was dose-dependent. Furthermore, CHIKV replication was completely inhibited in the murine serum, spleen, liver and brain. This suggests that the mouse IFN-α gene was constitutively expressed and the protein was transported via the bloodstream to these organs where it conferred protection against CHIKV replication. To further quantify and confirm the raised levels of IFN-α protein in the different organs, ELISA could be performed on the harvested tissues. Despite the persistent presence of CHIKV in the limbs, viral load was significantly reduced with pre-treatment of mDEF201. Clinically, CHIKV primarily causes chronic rheumatic manifestations at the limbs [26–28]. Therefore, the virus might persist in high amount as this is the site where CHIKV replication is favored. Nonetheless, the physical morphology of the limb muscle tissues was much intact without any signs of CHIKV-induced damage alike the rest of the other tissues when mice were protected with mDEF201 prior to infection. The ability of CHIKV to replicate in skeletal muscle and consequently causing myalgia and myositis has been well characterized [29]. However, it is unclear if the muscle tissue damage seen in CHIKV infections is caused directly by viral replication or is a result of inflammatory responses [29]. From our results, we hypothesize that tissue damage is caused by cytokines and chemokines released at the site of infection as a result of the inflammatory response mediated by viral replication in muscle cells during the acute phase of CHIKV infection. However, pre-treatment with mDEF201 can alleviate the extent of immune response and accelerate the elimination of CHIKV. Therefore, the muscle tissue integrity was less affected despite relatively high viral load in the limbs.

In summary, it could be postulated that the inhibition of CHIKV replication by mDEF201 at the various tissues maintained their morphological integrity and protected the mice against the onset of CHIKV pathogenesis, which lead to the observed 100% survival rate.

In addition, mDEF201 displayed some extend of therapeutic effect against CHIKV infection when initiated early during the acute infection by CHIKV. The conferred protection was reduced when mDEF201 was administered late (12 hours post infection) and was completely lost by 24 hours post infection. The suppression of CHIKV load in the various tissues was also observed to decline as the disease progressed with time. It was previously reported that upon successful viral RNA replication, the nonstructural protein 2 of CHIKV is responsible for inhibiting the host IFN induced signaling pathway [15,30]. This time-dependent therapeutic activity of mDEF201 was also observed in other studies performed on CHIKV as well as several other viruses [22,31,32]. Nevertheless, mice in this study were infected with lethal doses of CHIKV. During a natural infection, much lesser virus is transferred from the bite of a mosquito. Hence, the limited therapeutic efficacy of mDEF201 observed might be improved in clinically infected patients whose disease progressions are less severe. Further studies are warranted to determine the therapeutic activity of mDEF201 against CHIKV infections in the clinical setting.

Several other studies have reported that the adenovirus vector driven constitutive expression of IFN could inhibit viruses from different families, including the Western and Venezuelan equine encephalitis viruses, SARS and human enterovirus A71 [17–20]. These data indicate that mDEF201 could potentially be used as a broad-spectrum antiviral.

In conclusion, this study presented that mDEF201 is a highly effective prophylactic agent against systemic CHIKV infection *in vivo*. mDEF201 has immense clinical potential as a single dose could protect individuals at high risks during an outbreak. In addition, mDEF201 could also potentially be used to treat CHIKV infections especially during the acute phase. Additional work is required to determine the long-term prophylactic activity over the course of weeks and the therapeutic efficacy in a natural infection.

## Supporting information

S1 Table Mice clinical scoring system in CHIKV BALB/c mice model. The mice were assessed daily by the mouse clinical scoring system to determine the severity of clinical symptoms of the CHIKV infection.
(DOCX)
