# Supplementary material for: Adenovirus vectored IFN-α protects mice from lethal challenge of Chikungunya virus infection
Source: PLoS Negl Trop Dis. 2020 Dec 3;14(12):e0008910. doi: 10.1371/journal.pntd.0008910 (PMC7738163; doi:10.1371/journal.pntd.0008910)
Supplement: S1 Table — The mice were assessed daily by the mouse clinical scoring system to determine the severity of clinical symptoms of the CHIKV infection. (DOCX) [file pntd.0008910.s001.docx]

| Score | 0 | 1 | 2 | 3 |
| --- | --- | --- | --- | --- |
| Activity | Normal | Isolated, lethargy, abnormal posture | Huddled, inactive or overactive | Moribund or seizuring |
| Breathing | Normal | Rapid,  shallow | Rapid,  abdominal | Laboured, |
| Movement | Normal | Slight incoordination | Single limb dragging/  paralysis | Staggering, paralysis, multiple limb dragging |
| Body Weight | Normal(Gaining weight 0.5-1g/day) | Stopped or markedly reduced growth(5% loss over 24h) | Weight loss>15% or up to 10% in 24h | Weight loss>20% or >10% over 24h |
